# Cytotoxicity Analysis for the Hydroxyl Functionalized MWCNT Reinforced PMMA Nanocomposites in Oral Squamous Carcinoma (KB) Cells

**DOI:** 10.3390/polym15051192

**Published:** 2023-02-27

**Authors:** Vijay Patel, Unnati Joshi, Anand Joshi, Tarun Kumar Upadhyay, Lamya Ahmed Al-Keridis, Mohd Saeed

**Affiliations:** 1 Department of Mechanical Engineering, Parul University, Vadodara 391760, Gujarat, India; 2 Department of Mechatronics Engineering, Parul University, Vadodara 391760, Gujarat, India; 3 Department of Biotechnology, Parul Institute of Applied Sciences and Centre of Research for Development, Parul University, Vadodara 391760, Gujarat, India; 4 Biology Department, Faculty of Science, Princess Nourah Bint Abdulrahman University, P.O. Box 84428, Riyadh 11671, Saudi Arabia; 5 Department of Biology, College of Sciences, University of Hail, Hail 34464, Saudi Arabia

**Keywords:** nanocomposite, cytotoxicity, multiwall carbon nanotubes, PMMA, KB cells

## Abstract

In this particular research study, a unique three-dimensional mixing technique was used to incorporate multi-walled carbon nanotubes (MWCNTs) into polymethyl methacrylate (PMMA), and the KB cell line was used in the analysis of cytotoxicity, apoptosis detection, and cell viability using the MTT assay protocol. At low concentrations (0.001 to 0.1 g/mL), these results showed that the CNT did not seem to cause cell death or apoptosis directly. It increased lymphocyte-mediated cytotoxicity against KB cell lines. This was demonstrated by the fact that the CNT increased the time it took for KB cell lines to die. In the end, the unique three-dimensional mixing method solves problems such as clumping and uneven mixing that have been written about in the relevant literature. Phagocytic uptake of MWCNT-reinforced PMMA nanocomposite by KB cells leads to oxidative stress and apoptosis induction in a dose-dependent manner. The cytotoxicity of the generated composite and the ROS (reactive oxygen species) it produces may be controlled by adjusting the MWCNT loading. The conclusion that can be drawn from the studies to date is that it could be possible to treat some types of cancer using PMMA that has MWCNTs incorporated into it.

## 1. Introduction

Researchers in engineering and biomedicine are interested in carbon nanotubes (CNTs) because of how they are made and what they can do. These structures and properties include high aspect ratios, sizeable surface areas, a wealth of surface chemical functionalities, and size stability on the nanoscale. They have a lot of potential as agents and carriers for cancer treatment, which is why the field is receiving so much attention. Since the 1960s, the material known as polymethyl methacrylate (PMMA) has been put to use in a variety of biomedical fields, including orthopedics and dentistry. PMMA is gradually being phased out in favor of other materials with superior mechanical properties. PMMA, on the other hand, has room for improvement in its bioactivity. Nanotechnology is becoming more critical in biomedicine, where it can be used in many ways. This has been a driving force behind the development of new nanomaterials. Since the discovery of carbon nanotubes (CNTs), the use of nanocomposite materials has been proliferating in highly advanced industrial settings [1]. Concerns regarding the toxicity and biocompatibility of CNTs have been raised in response to the rise in the number of possible uses of these nanotubes in biomedical fields. Because the immune system usually plays a role in tissue damage during pathogenesis, it is essential to determine if CNTs can cause cytotoxicity by affecting how the immune system works. Because of their exceptional mechanical, electrical, and optical properties, they have drawn the attention of scientists and engineers from academic institutions as well as private industry for use in a wide variety of technological applications, including those in the fields of automobiles and aerospace, biomedicine, packaging, electrical and electronic devices, and drug delivery, among others [2,3,4]. The structure of a CNT is made up of graphite layers rolled around the central axis. These layers have lengths and diameters that range from micrometers to nanometers, according to the number of layers of rolled-up graphene sheet in a coaxial orientation. Carbon nanotubes come in three different kinds: single-walled carbon nanotubes (SWCNTs), double-walled carbon nanotubes (DWCNTs), and multi-walled carbon nanotubes (MWCNTs) [5]. The processes of chemical vapor deposition (CVD), physical vapor deposition (PVD), arc discharge, and laser ablation can all be used to make carbon nanotubes. CVD is the most commonly used method because it is easy to deploy and can be scaled up [6]. Not only does the way the material is made have a significant effect on its properties, but so does the size of the particles used to make it stronger [7]. A large quantity of published material is accessible, each containing a unique mixing ratio, technique of synthesis, mixing process, and variety of components.

Epoxy resin was synthesized by Hallad et al. using a tiny concentration of 0.4 weight percent MWCNT, and the results showed a significant increase in the material’s mechanical characteristics [8]. Nanocomposites were produced by melt-mixing three unique thermoplastic matrices in MWCNTs. These matrices were polypropylene (PP), polycarbonate (PC), and thermoplastic polyurethane (TPU). The filler percentages were one, three, and five weight percent. According to Quadrini [9], there is no question that the interaction between these matrices and MWCNTs results in a positive outcome for the performance of the composite. The reinforcement of polyetheretherketone (PEEK) with the addition of carbon nanotubes (CNTs) was prepared by a powder mixing process. After the material was evaluated for its electrical, thermal, and tensile properties, it was found that the addition of CNTs improved all of these properties [10]. Cancer cell lines such as the human breast adenocarcinoma cell line (MCF7) [11], human hepatocarcinoma, human astrocytoma and lung carcinoma, human neuroblastoma cells, or the C6 rat glioma cells have been shown to be susceptible to the cytotoxic effects of CNT in vitro. Topotecan (TPT) [12], mesoporous silica nanoparticles (MSNs) [13], metformin (MT) [14] and their combinations with polymer nanocomposites were analytically evaluated by MTT assay by various concentrations (0.05–2 g/mL) of drug. With respect to cellular uptake efficiency and therapeutic mechanisms as in vitro and in vivo models, authors [15] examined the nanocarrier-mediated inhibitory effects of topotecan (TPT) and quercetin (QT) on triple-negative breast cancer (TNBC) (MDA-MB-231) and multi-drug-resistant (MDR) type breast cancer (MCF-7) cells. This was done in order to determine the functionalized single-walled carbon nanotube conjugated with chitooligosaccharide, also known as f-SWNT-COS, which is utilized in the manufacturing of drug delivery systems. According to the findings, the f-SWNTs-COS-GTX-p53 is the most effective delivery vehicle for producing regulated release and improving cytotoxicity in human cervical cancer (HeLa) cells. This was determined by analyzing the data. In addition, these devices have the potential to be used in the distribution of additional anti-cancer drugs that are now on the market [16]. The results of the cytotoxic tests performed on tumor cells using CNTox, CNT-Dox, and CNT-FITC in vitro (2-D and 3-D cultures), as well as in vivo using the Balb2/c mouse model, demonstrate that doxorubicin may be immobilized on the surface of CNTs and released from that surface in a controlled way. When compared to free Dox, the cytotoxic impact of CNT-Dox, which is an immobilized form of Dox, is significantly reduced. The year 2018’s “Perepelytsina” using oxidized multi-walled carbon nanotubes (MWCNT-COOH) functionalized with biocompatible polymers as a viable drug carrier led to the development of an effective therapeutic silibinin (SB) for the treatment of cancer. The MTT experiment showed that coated MWSB nanocomposites on 3T3 cells were extremely biocompatible at concentrations as high as 100 g/mL. This finding provides proof that MWSB has a lower potential for cytotoxicity [17]. The impacts of polymer-functionalized CNTS on anticancer drug delivery systems, as investigated by in vivo and in vitro behaviors, as well as the implications of the same on toxicity, are reviewed in this article. The findings suggest that CNT-based Nano carriers may provide unique prospects for the early identification and treatment of cancer, and they also reveal that functionalized CNTs were not dangerous when delivered to mice [18]. According to Rizwan et al, who presented cytotoxic and genetic responses to liver cancer cells and their examination of the viability of grown cells using the MTT assay in conjunction with the effects of doses of CNTs and HA powder, the growth of cancer cells can be inhibited by polymeric materials in a dose-dependent manner [19]. In research that was conducted by Nasirzadeh and colleagues, the authors explored how MWCNTs influence the cellular toxicity of human alveolar epithelium. The A549 cells were allowed to mature and then treated to varying concentrations of MWCNTs in three consecutive time periods. The cellular viability of the sample was evaluated using the MTT assay, which is a colorimetric method for measuring tetrazolium. The investigation revealed that the cytotoxicity of MWCNTs on human alveolar epithelial cells is proportional to the exposure dose as well as the length of that exposure [20]. In order to make chemotherapy more effective, Gonzalez et al. looked into the possibility of using MWCNTS in the formulation of drug delivery systems. They contrasted the impact of 5-fluorouracil (5-FU) with that of an antimetabolite medication that was physiosorbed onto MWCNTs. 5-FU is a therapy for various different types of cancer. According to the findings of our research, the overall efficacy of the medicine may be significantly improved by a factor of 50% if it is given intratumorally while also being linked to MWCNTs in models of solid tumors that are tested in vitro and in vivo [21]. MCF-7 is a human breast cancer cell line that was used in the study by Bellucci et al. to explore the effects of MWCNTs, MWCNTs-COOH, and MWCNTs-OH at concentrations of 0.1 mg/mL. According to the findings, the cytotoxic effects of any type of MWCNTs may be attributed to both a rise in the rate of apoptotic cell death and a fall in the rate of proliferation [22]. Using the MTT, WST-1, Hoechst, and oxidative stress tests, Vittorio and colleagues looked at the effects that different physicochemical qualities of MWCNTs have on the toxicity and biocompatibility of MWCNTs when used with neuroblastoma cells. Concentrations of 5–10 mug/mL MWCNTs appear to be advantageous for investigations on the invention and fabrication of synthetic MWCNT Nano vectors for gene and pharmacological treatment against cancer, as indicated by our research [23]. Maria et al. investigated the cytotoxic and genotoxic effects of carbon black, as well as single and multi-walled carbon nanotubes (SWCNTs and MWCNTs). The effects of MWCNTs with carboxylic acid functionalization (MWCNTs-COOH) and unprocessed MWCNTs (p-MWCNTs) on RAW264.7 cells were significantly different from those of unprocessed MWCNTs. These differences were observed in terms of cell viability, phagocytic uptake activity, cytokine production (IL and TNF), and intracellular reactive oxygen species (ROS) [24]. It was discovered through research carried out by Sun and colleagues that CNT is responsible for mediating the in vitro cytotoxicity that is caused by human lymphocytes. The findings showed that although low doses of CNT (0.001 to 0.1 mg/mL) did not directly result in cell death or apoptosis, they did boost lymphocyte-mediated cytotoxicity against a number of human cell lines. This was shown by the fact that the CNT increased the number of human cell lines that were killed. In this investigation, our primary focus was on integrating hydroxyl-functionalized CNTs because this modification improves the dispersibility of the CNTs and results in homogenous mixing. After conducting research on the morphological features of CNT-incorporated Polymethyl methacrylate (PMMA) (containing 0.1 to 1 weight% CNT), the ensuing mechanical properties, and cytotoxicity evaluations, we came to the following conclusion: We looked into the effects of COOH and OH functionalized multi-walled carbon nanotubes on the KB cell line in order to determine whether or not COOH or OH functionalized MWCNTs caused an increase in toxicity in comparison to pure (i.e., non-functionalized) carbon nanotubes.

PMMA is a popular and ideal biomaterial for various dental applications due to its low density, aesthetics, lower cost, easy processing, and tailorable physical and mechanical characteristics. PMMA is employed for prosthetic dental applications, such as the creation of artificial teeth, denture bases, orthodontic retainers, crowns, and for the repair of dental prostheses, as mentioned by Zafar in his published review report [25]. Hasan et al. [26] discussed prosthodontic applications of PMMA and its drawbacks with respect to its mechanical properties. Due to its weak mechanical attributes (flexural and impact strengths) and propensity to deteriorate over time as a result of water sorption, the addition of metal wires, different nanofibers, and rubber-based copolymer will increase the mechanical properties of PMMA. Although it has been demonstrated that adding carbon/graphite fibres (CFs) to PMMA resins may significantly improve the flexural and impact strengths of dentures made of acrylic resin, this material is not frequently employed because of its coloration. The addition of glass fibres (GFs) to PMMA resins for denture strengthening not only has good mechanical capabilities but also outstanding cosmetic qualities. In spite of the clear advantages of fibre reinforcing for PMMA dentures, the practical adoption of these appliances is still constrained because of their high price and challenging synthesis process.

## 2. Materials and Methods

### 2.1. MWCNT and PMMA

Hydroxyl functionalized MWCNTs with a diameter of 10–15 nm and length of 10–20 µm were procured from Platonic Nanotech Pvt LTD, India. Polymethyl methacrylate (PMMA) as a fine powder was purchased from N. Shashikant& Co., Mumbai, India. The properties of PMMA and MWCNTs are as shown in Table 1.

### 2.2. Functionalization of Carbon Nanotubes

Because they are insoluble and have a low dispersibility, pure CNTs have a limited range of applications due to their poor solubility, which causes them to agglomerate, and their poisonous nature. Zahra et al. [28] state that it is of the utmost importance to find a solution to these problems and enhance their natural qualities through surface modification. According to the research that was conducted, surface-functionalized carbon nanotubes are significantly less hazardous than pure carbon nanotubes [29]. These methods may be simply broken down into two categories: chemical (covalent) functionalization and physical (noncovalent) functionalization [30]. Both of these categories refer to interactions between active materials and CNTs.

The benefits of using MWCNT that has been functionalized [28,29,30].

- Superior Conductivity
- Chemical Inertness
- Enhancement of Flexibility
- Biocompatibility
- Water Dissolution rate
- Heat Resistance
- Large Surface

### 2.3. Synthesis, Testing and Characterization

#### 2.3.1. 3D Mixture

**Figure 1:**
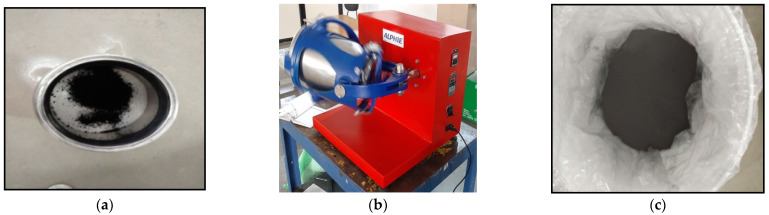
Synthesis of polymer nanocomposite (**a**) PMMA/MWCNT Powder (**b**) PMMA/MWCNT in 3D Mixture (**c**) Final Mixture Product.

PMMA and OH-MWCNTs were obtained in the form of a powder and stored in an airtight container. During the 3D mixing process, the powder was mixed not by rotation but rather by an inversion motion as shown in Figure 1. A total of three distinct weight percentages of OH-MWCNTs were included in the mixing batches (0.1, 0.5, and 1 wt%). The homogenous mixture of PMMA and OH-MWCNT was warmed at 80 degrees Celsius for eight hours in order to eliminate any moisture content. After this, the molding process was continued in order to prepare specimens for mechanical testing.

#### 2.3.2. Injection Molding Machine

**Figure 2:**
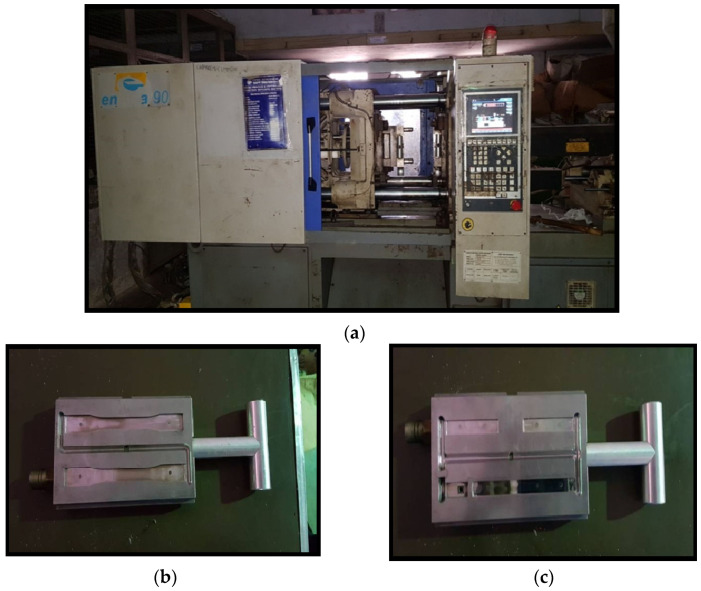
(**a**) Injection Molding Setup (**b**,**c**) Dies for tensile and flextural specimen.

To prepare the test samples, the mix of uniformly dispersed MWCNT and PMMA is processed by a twin screw extruder, followed by an injection molding process. The melted material flows from barrel to mold, at a high temperature, which later cools down and solidifies to the desired shape and size. The injection molding machine and dies for test specimens are shown in Figure 2a–c, respectively.

#### 2.3.3. Universal Testing Machine/Tensile Testing

**Figure 3:**
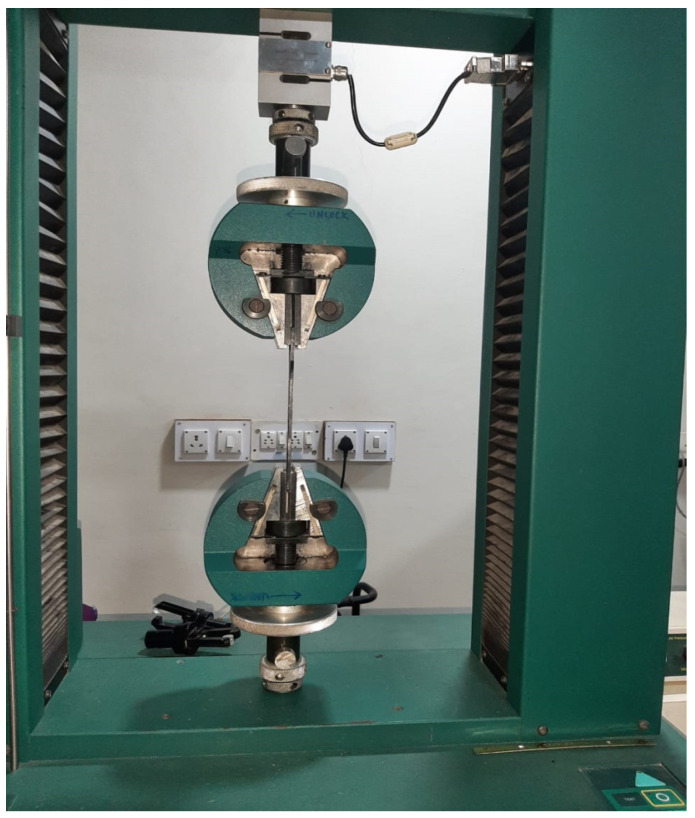
Universal Testing Machine.

A tensile test of the prepared nanocomposite specimen was performed on an universal testing machine made of Tinius Olsen to study the effect of carbon nanotubes loading on the mechanical properties of polymer as shown in Figure 3. Tests were carried out on five samples according to the ASTM D 3039 test method at a cross-head speed of 0.01 mm/min using a 10 KN load cell.

#### 2.3.4. Three Point Bending Test/Flextural Testing

**Figure 4:**
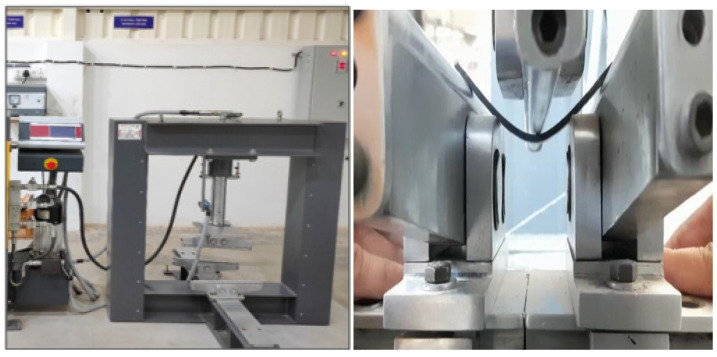
Flexural Test Setup.

A three-point loading system utilizing center loading on a simply supported beam method was used for flexural testing of the prepared specimen as shown in Figure 4. Flexural strength provides the maximum stress on the outer surface of a flexure test specimen corresponding to the peak applied force prior to flexural failure [31]. The flexural modulus is defined as the ratio of the stress range to the corresponding strain range for a test specimen loaded in flexure.

#### 2.3.5. Scanning Electron Microscopy

**Figure 5:**
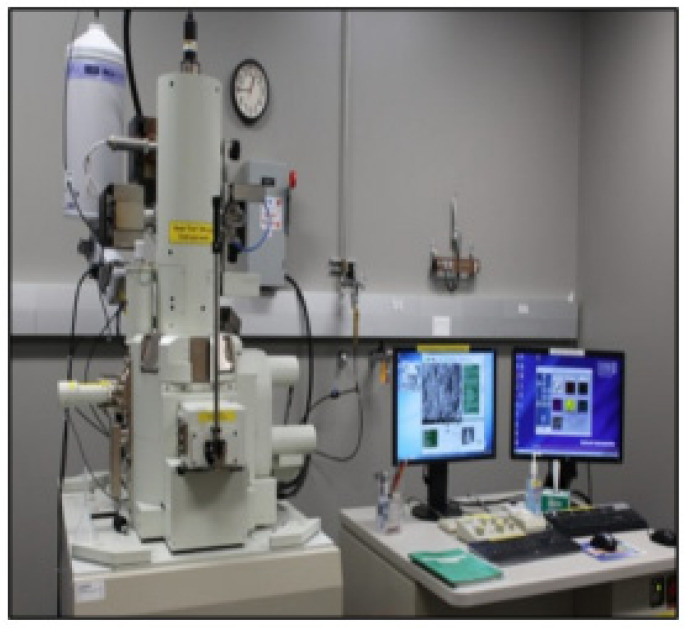
Setup of Scanning Electron Microscopy.

Scanning electron microscopy (SEM) was performed on small pieces taken from the injection molded samples using a low voltage SEM (JEOL JSM-7010LA) as shown in Figure 5. The most commonly utilized method for examining the microstructure of nanocomposites is to look at the fractured surfaces.

### 2.4. Cell Culture and Maintenance 

For the purpose of this research, the oral squamous carcinoma (KB cell line) was generously donated by the National Centre for Cell Science (NCCS, Pune, India). The Dulbecco’s modified Eagle medium (DMEM), the fetal bovine serum (FBS), the antibiotic, and the antimitotic solution were all acquired from Gibco^TM^ (Thermo Fisher Scientific, Waltham, MA, USA). In a humid environment at 37 degrees Celsius and 5% carbon dioxide, the viability of the cells was maintained. In order to improve the quality of the DMEM cell culture medium, 10% FBS and 1% antibiotic and antimitotic solution were added.

### 2.5. In Vitro Uptake Study 

It was seeded and cultivated overnight at 37 °C with 5% carbon dioxide on a 96-well plate with adherent 0.1 × 10^6^ KB cells in each well. The cells were then subjected to fluorescent particles by replacing the culture media in the wells with hydroxyl-functionalized MWCNT Reinforced PMMA Nanocomposites that were distributed in HBSS. After being exposed for 0–10 min, the cells were observed under a fluorescence microscope (Thermofisher Scientific, Waltham, MA, USA) to see if they contained any particles that emitted a fluorescent light.

### 2.6. Reactive Oxygen Species Generation through DCHF-DA Assay 

In order to examine the formation of reactive oxygen species (ROS) in the KB cell line, 1 × 10^5^ KB cells were seeded in each well of a 12-well plate with DMEM overnight at 37 degrees Celsius. After a first round of incubation lasting 24 h, the cells were then subjected to doses of 50, 100, and 150 mg/mL of hydroxyl-functionalized MWCNT reinforced PMMA nanocomposites before undergoing a second round of incubation lasting 24 h. Using the H2DCFDA dye, it was possible to monitor cellular ROS generation after the incubation time had concluded. After adding H2DCFDA at a concentration of 10 mm to the cells for a period of 15 min, microscope pictures of ROS generation were obtained throughout this time.

### 2.7. Cellular Reactive Oxygen Species Generation upon Hydroxyl Functionalized MWCNT Reinforced PMMA Nanocomposites

ROS production is a major contributor to the development of chronic illnesses such as cancer. The production of cellular ROS is a process that is part of the living system and is used to maintain and regulate the metabolism. In normal cells, a mechanism that scavenges free radicals can control the creation and clearance of reactive oxygen species (ROS), but cancer cells create a large number of ROS in order to control the increased metabolic activity that they undergo. This increased creation of ROS has the potential to cause damage to genetic information, proteins, and lipids, which may ultimately lead to apoptosis in the cells. Chemotherapeutic drugs generate a significant amount of ROS, which inhibit the progression of cancer.

### 2.8. Apoptosis Detection through Dual Staining with Hoechst and PI Staining 

A dual staining of KB cells experiment was performed on 105 cells/well that were planted in 12-well plates and treated with doses of 50, 100, and 150 mg/mL of hydroxyl functionalized MWCNTs. This was done in order to evaluate the morphology of the apoptotic cells. Nanocomposites of reinforced PMMA were incubated with a 24 h time window. After incubation, the treated cells were stained with a concentration of 1 mg/mL for 15 min. Morphological changes were documented through the use of microscopic photography.

## 3. Results and Discussion

### 3.1. Mechanical Characterization

The mechanical properties of the PMMA/OH-MWCNTs composite were measured using a universal test with ASTMD3039 and ASTM D7264, respectively. Five specimens for each combination were prepared and tested for mechanical properties with the mentioned standard. The mean results of the tensile and flexural test are shown in Table 2.

**Figure 6:**
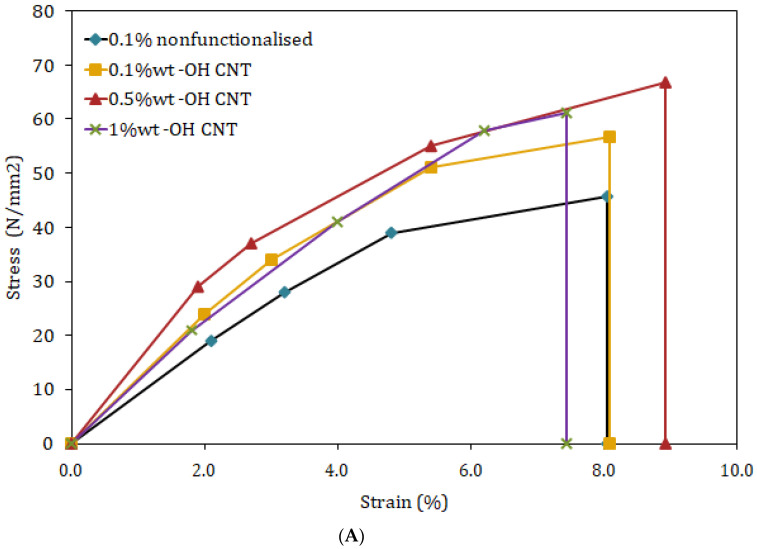

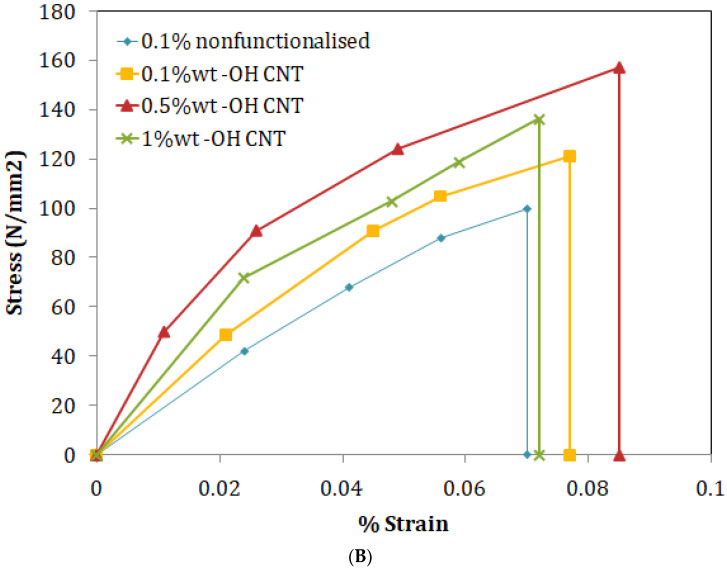
(**A**) Tensile test results of 0.1 wt%, 0.5 wt% and 1.0 wt% loading of hydroxyl functionalized MWCNT in PMMA (**B**) Flexural results of 0.1 wt%, 0.5 wt% and 1.0 wt% loading of hydroxyl functionalized MWCNT in PMMA.

According to the findings of the experiment, there is an increase in tensile strength of 27.6%, 48.33%, and 37.97%, correspondingly, for 0.1, 0.5, and 1.0 wt% OH-MWCNT in PMMA compared to neat PMMA as shown in Figure 6A. When compared to clean PMMA, the flexural strength is improved by 18.74%, 53.89%, and 33.66%, respectively, when 0.1, 0.5, and 1.0 wt% of OH-MWCNT are present in the PMMA as shown in Figure 6B. Both the tensile strength and the flexural strength of the MWCNT/PMMA composite material improve with increasing weight percentages of MWCNT up to 0.5 wt%, beyond which point they begin to decline. The cause of this variation may be a rise in the filler content, the high viscosity of the polymer in its molten form, which makes it harder, and a lack of flexibility, which causes a further decline in characteristics [28].

### 3.2. Morphological Characteristics 

**Figure 7:**
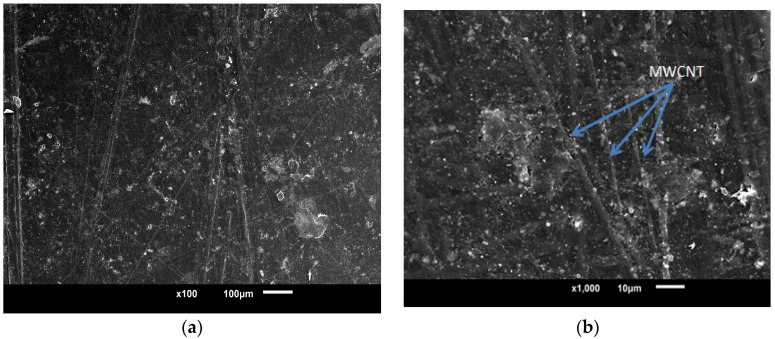
Scanning Electron Micrograph (**a**) SEM Micrograph (**b**) SEM micrograph at high magnification.

In order to investigate the dispersion and distribution of CNTs inside the generated nanocomposites, a scanning electron microscopy instrument manufactured by Jeol Ltd. (Akishima, Tokyo, Japan) and designated as a JSM-6010LA was utilized. The SEM pictures, as shown in Figure 7, provide for clear observation of the uniform distribution of functionalized nanotubes throughout the sample. Additionally, it was observed that there is no breaking of MWCNTs throughout the processing, which shows that the 3D mixing procedure led to an excellent result in the processing of nanocomposites.

### 3.3. In Vitro Phagocytic Uptake Studies

**Figure 8:**
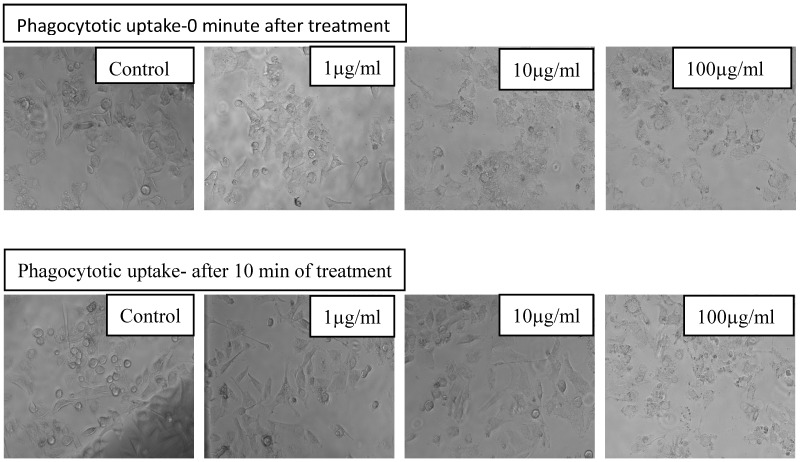
Phagocytic uptake of hydroxyl functionalized MWCNT reinforced PMMA nanocomposites upon exposure to KB cells in the concentration range of 1–100 μg/mL after 0 and 10 min exposure.

A phagocytic assay was used to analyze the interaction of MWCNT-reinforced PMMA polymer nanocomposites with KB cells. The mixture at a concentration of 1 to 100 μg/mL was used for phagocytic uptake experiments with 0–10 min of exposure and their micrographs were captured as shown in Figure 8. 

### 3.4. In Vitro Cell Viability Assay 

An experiment using tetrazolium dye (3-[4, 5-dimethylthiazol-2-yl]-2, 5-diphenyltetrazolium bromide [MTT]) was carried out to assess the inhibition of cell growth in order to determine the cell survival of the hydroxyl functionalized MWCNT-reinforced PMMA nanocomposites. This was done in order to determine whether or not the hydroxyl functionalized MWCNTs have cytotoxicity. In a 96-well plate, 1 × 10^6^ KB cells were seeded into each well of the plate. Subsequently, the cells were placed in an incubator at 37 °C. and 5% CO_2_ for 24 h and subjected to hydroxyl functionalized MWCNT-reinforced PMMA nanocomposites at various concentrations (0–100 g/mL). Following this, 10 L of the MTT solution containing 5.0 milligrams per milliliter was added to each well. After adding the MTT, the plates continued to be incubated at 37 degrees Celsius for a further 4 h. Following the removal of the supernatant, 150 liters of DMSO were added in order to thoroughly dissolve the crystals. The absorbance at 490 nm, which is proportional to the number of metabolically active cells, was evaluated by using a multimode ELISA plate reader (EXL-800; BioTek Instruments, Winooski, VT, USA). The proportion of viable cells may be calculated as follows: (Abs. sample/Abs. control) multiplied by 100 equals the percentage of viable cells. This formula was used to determine the viability of the cells. It was changed to reflect a survival rate of 100% for the untreated controls. Cell death occurs when the percentage of viable cells falls below 100%.

#### Cell Viability 

**Figure 9:**
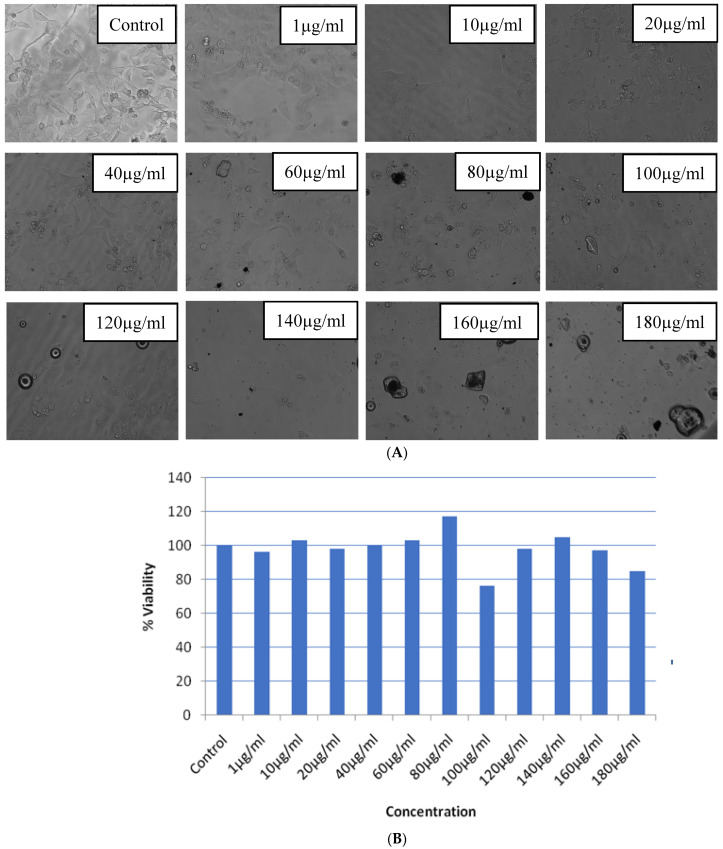
Cell viability/cytotoxic potential of hydroxyl functionalized MWCNT-reinforced PMMA nanocomposites upon 24 h exposure to KB cells in the dose-dependent manner of a concentration range of 1–180 μg/mL (**A**) Microscopic graph (**B**) Cumulative histogram.

The MTT test was utilized in order to evaluate the possible applications of hydroxyl-functionalized MWCNT-reinforced PMMA nanocomposites. Figure 9A,B demonstrates that the growth of the cancer cell line KB was significantly suppressed after it was treated for 24 h to varying dosages (50–200 mg/mL) of hydroxyl functionalized MWCNT-reinforced PMMA nanocomposites. The IC50 concentration against the KB cells was found to be 136 mg/mL, and concentrations both below and above IC50 were used for further testing at these levels.

### 3.5. Cellular Reactive Oxygen Species Generation upon Hydroxyl Functionalized MWCNT Reinforced PMMA Nanocomposites

ROS production is a major contributor to the development of chronic illnesses such as cancer. The production of cellular ROS is a process that is part of the living system and is used to maintain and regulate the metabolism. In normal cells, a mechanism that scavenges free radicals can control the creation and clearance of ROS, but cancer cells create a large number of ROS in order to control the increased metabolic activity that they undergo. This increased creation of ROS has the potential to cause damage to genetic information, proteins, and lipids, which may ultimately lead to apoptosis in the cells. Chemotherapeutic drugs generate a significant number of ROS, which inhibits the progression of cancer.

**Figure 10:**
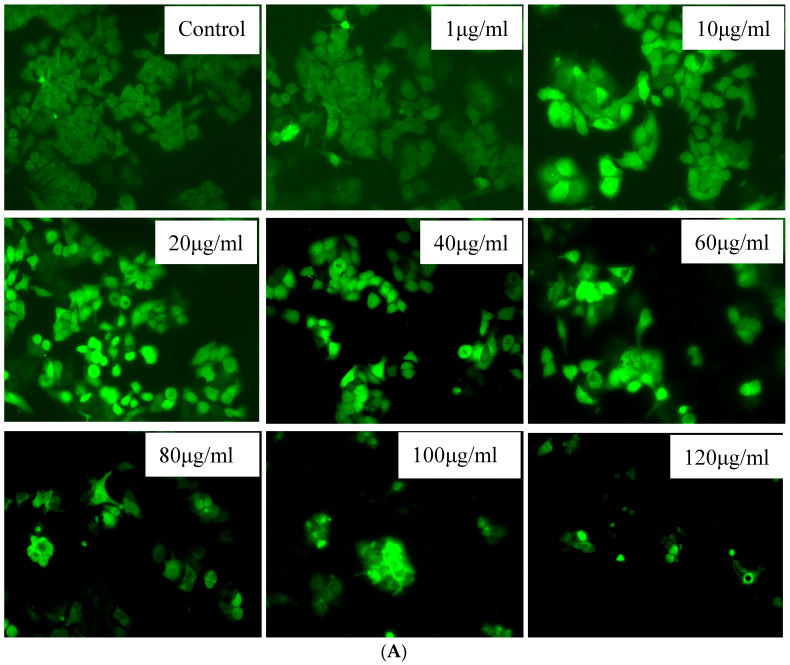

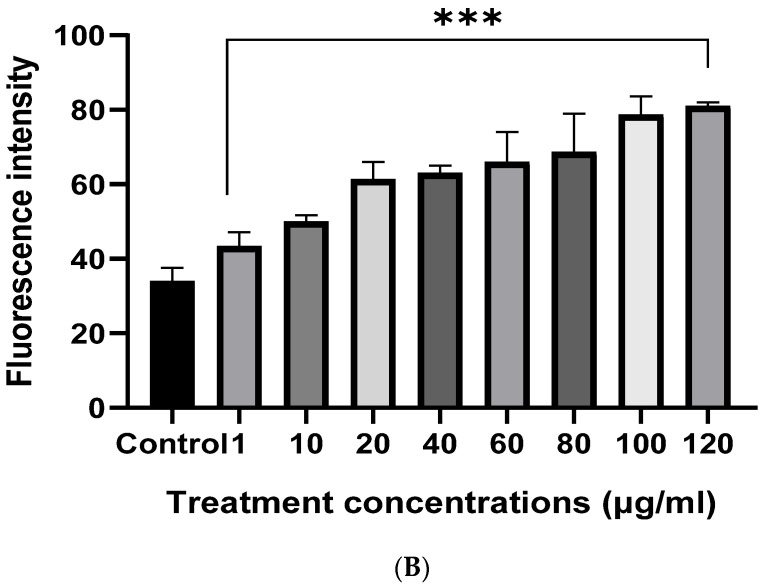
Effect of MWCNT-reinforced PMMA nanocomposites with hydroxyl functionalization on intracellular ROS levels in the KB Cell (**A**) Fluorescence microscopic images with green fluorescence demonstrating intracellular ROS generation in KB cells using DCFH-DA labelling (**B**) GraphpadPrism version 8 and intracellular ROS pictures were evaluated using ImageJ software. *** *p* < 0.001 denote highly significant data.

In order to determine the role that ROS production plays in cancer cells, we tested hydroxyl-functionalized MWCNT-reinforced PMMA nanocomposites in KB cells at concentrations ranging from 50 to 150 mg/mL, as shown in Figure 10A. As can be observed in Figure 10B, there was a discernible increase in the formation of ROS as concentrations increased. When analyzed using a one-way ANOVA utilizing the GraphPad Prism 8.0 software, the findings were found to be statistically significant, with a *p*-value of 0.001 or below in every case (*p* < 0.001). The generation of intracellular ROS was shown to substantially increase after KB cells were treated with hydroxyl functionalized MWCNT-reinforced PMMA nanocomposites. The formation of ROS in KB cells was significantly boosted when they were treated with hydroxyl-functionalized MWCNT-reinforced PMMA nanocomposites (a specific condition). In the photomicrographs, the vivid green fluorescence that was induced by the formation of ROS in the control KB cells could be seen very clearly, as shown in Figure 10B. KB cells that had been treated with hydroxyl functionalized MWCNT-reinforced PMMA nanocomposites could be observed against a background with a very faint green fluorescent signal. Following treatment of KB cells with hydroxyl functionalized MWCNT-reinforced PMMA nanocomposites (range of concentration), enhanced DCF dye fluorescence was seen in the nucleus of the cells, demonstrating an increase in ROS generation.

### 3.6. Hoechst and PI Staining Assay 

**Figure 11:**
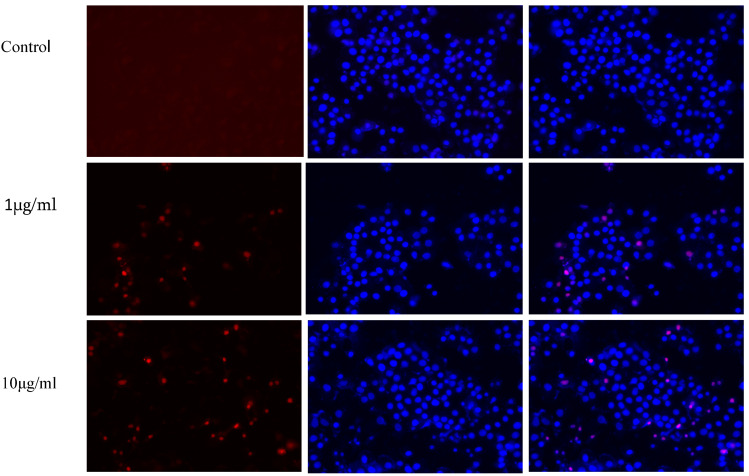
Dual staining of KB cells with Hoechst (blue colour) and PI (red colour) upon 24 h exposure to different concentrations of nanocomposite. Control cells exhibited no dual staining and in the treated cells, the number of dead cells increases while increasing the concentration.

Under a fluorescent microscope, apoptotic cells that had DNA that was both clumped together and fragmented could be observed after being labelled with Hoechst and PI staining dye, as shown in Figure 10. HeLa cells were stained after receiving treatment for 24 h at dosages ranging from 50 to 150 mg/mL and then being stained, as shown in Figure 11 During this particular test, treated cells were shown to undergo visible morphological changes, while untreated cells were found to contain their nuclei intact. All of the treatments were found to be significant after performing a one-way ANOVA using the GraphPad Prism 8.0 statistical software package as shown in Figure 11, setting the significance threshold at *p* value 0.05, and performing a quantitative estimate using the ImageJ software.

Increases in fluorescence intensity with increasing treatment concentrations indicate clearly discernible DCF fluorescence in cancer cells treated in a variety of concentration-dependent ways using hydroxyl functionalized MWCNT-reinforced PMMA nanocomposites. ANOVA with DMRT was used to compare the results of independent studies denoted by asterisks. In comparison with the control, all of the results were found to be statistically significant. Statistically different results from control * *p* < 0.05 are indicated by an asterisk.

Propidium Iodide-stained cells were observed in red fluorescence and Hoechst-stained cells were observed under blue fluorescence. Combined images showed that apoptosis of the cells was increased with the increase in nanocomposite concentration that was indicated by the increased pink (combined) color fluorescence intensity.

## 4. Conclusions

In the current study, we examined the extrusion, injection molding, and plastics-processing processes used to create polymer nanocomposites and evaluated their mechanical and cytotoxic properties. The PMMA/OH-MWCNT composite leads to apoptosis induction in the malignant KB cells in the dose-dependent manners. The main findings of the investigations conducted and discussed in this article may inspire fresher concepts for improving cancer treatment through composite materials disease. The same technology was used to test a variety of polymer combinations, but due to non-homogeneous mixing, the ones stated above are replacing traditional drug delivery systems due to their important advantages, such as minimal harm to healthy cells and a lack of side effects. For cancer treatments, applying external or internal stimulation is strongly advised. They are a strong impulse for the continuation of research in this area. As a result, we can conclude that MWCNT-reinforced PMMA has the potential to inhibit the growth of cervical cancer cells. This material can now be further explored in order to reveal its full anticancer potential.

## Figures and Tables

**Table 1:** PMMA and MWCNT Properties [Adapted from Ref. [27]].

**PMMA Properties**	
**Properties**	Description
**Color**	White
**Particle Size**	48 μm
**Tensile Strength**	45 MPa
**Young Modulus**	2855 MPa
**Density**	1.18 g/cm^3^
**MWCNT Properties**	
**Color**	Black Powder
**Diameter**	10–20 nm
**Length**	3–8 μm
**Purity**	>98 wt% (MWCNT)
**OH Content %**	2–4 wt%
**Surface Area**	90–350 m^2^/g
**Density**	2.6 g/cm^3^
**Young Modulus**	1.1 TPa

**Table 2:** Summary of Tensile and Flexural Test of 0.1 wt%, 0.5 wt% and 1.0 wt% loading of hydroxyl functionalized MWCNT in PMMA.

**Tensile Test Results**	
CNT %wt of MWCNT	Tensile Strength (N/mm^2^)
0.10 wt% non-functionalized MWCNT	43.67
0.1 wt% OH functionalized MWCNT	57.42
0.5 wt% OH functionalized MWCNT	66.75
1 wt% OH functionalized MWCNT	61.58
**Flexural Test Results**	
CNT %wt	Flexural Strength (N/mm^2^)
0.10% non functionalized MWCNT/PMMA	99.85
0.10% OH-functionalized MWCNT/PMMA	121.12
0.50% OH-functionalized MWCNT/PMMA	156.97
1.00% OH-functionalized MWCNT/PMMA	136.34

## Data Availability

All the data are mentioned in the manuscript.
